# Drug Utilisation and Off-Label Use on a German Neonatal Intensive Care Unit: A Retrospective Cohort Study and 10-Year Comparison

**DOI:** 10.3390/pharmacy8030173

**Published:** 2020-09-17

**Authors:** Carmen Geißler, Christopher Schulze, Sebastian Botzenhardt, Wolfgang Rascher, Antje Neubert

**Affiliations:** Department of Paediatrics and Adolescent Medicine, University Hospital Erlangen, Friedrich-Alexander-University Erlangen-Nürnberg (FAU), 91054 Erlangen, Germany; christopher.schulze@uk-erlangen.de (C.S.); sebastian@botzenhardt.de (S.B.); wolfgang.rascher@uk-erlangen.de (W.R.); antje.neubert@uk-erlangen.de (A.N.)

**Keywords:** off-label use, prescribing, NICU, European Paediatric Regulation

## Abstract

Pharmacotherapy of neonates is complex and marked to a large extent of off-label use. The implementation of the Paediatric Regulation (2007) gave hope for a change in the safety and efficacy for drugs used in neonatal intensive care units (NICU). This study investigates drug utilisation patterns and off-label use in a German neonatal intensive care unit (NICU) in 2014. A 12-months retrospective, observational cohort study was performed at the NICU of the University Children’s Hospital Erlangen, Germany. Licensing status was determined using the Summary of Product Characteristics (SmPC). Results are compared with a similar study conducted 10 years earlier. The study included 204 patients (57.8% male) (2004: 183) and 2274 drug prescriptions were recorded (2004: 1978). The drugs that were mostly prescribed were drugs for the nervous system (2004: 22.6%; 2014: 26.9%) and anti-infectives for systemic use (2004: 26.0%; 2014: 24.9%);34.3% (2004) and 39.2% (2014) of all prescriptions were off-label;62.7% of all patients received at least one off-label or unlicensed drug (2004: 70%). For 13 drugs, the licensing status changed either from off-label to label (n = 9) or vice versa (n = 4). Overall, there was no significant change neither in terms of the drugs used nor regarding their licensing status. Further studies are needed to validate these findings in a European context.

## 1. Introduction

Pharmacological treatment of neonates is a complex process and requires considerable expertise in neonatology and pharmacology [1]. Many organ functions such as the hepatic and renal metabolic and elimination capacity are not fully developed yet. Moreover, not only pharmacokinetics, but also pharmacodynamics in neonates differ from those in older children and adults [1,2].

In the neonatal intensive care unit (NICU) setting, this situation is even more complex due to severe diseases, a large amount of drugs prescribed and early gestational age with low birth weight [3,4]. Preterm neonates often require life support and suffer from organ immaturity. Term neonates on the ICU commonly suffer from congenital diseases and peri-/postnatal complications.

Safety and efficacy data on neonatal pharmacotherapy are scarce for many drugs. Up to 90% of neonates in intensive care receive drugs that are used in an unlicensed or off-label manner [5,6,7].

The Paediatric Regulation, which was implemented in the European Union in 2007, was introduced to reduce off-label use for children and to increase the clinical evidence in paediatric pharmacotherapy [8,9,10]. Various measures, like an extension of the patent protection, were adopted to reward the companies for their dedication. Furthermore, public funding programmes became available to also facilitate academic-driven paediatric research [11].

In 2004, a prospective, cohort-based observational study investigating the drug utilisation pattern and off-label/unlicensed drug use on an NICU was conducted at the University Children’s Hospital Erlangen, Germany [12].

Ten years later, seven years after the paediatric regulation came into force, this study was repeated to investigate the developments in drug utilisation on NICUs.

In this manuscript, we compare the results of these two studies and investigate the impact of the Paediatric Regulation with respect to the drugs used on this NICU and their licensing status.

## 2. Materials and Methods

### 2.1. Study Design

A retrospective cohort study was conducted and results were compared with the findings of a previously published cohort study.

### 2.2. Study Cohort

All preterm and term neonates admitted to the NICU at a University Children’s Hospital were enrolled between December 1st 2004 and October 31st 2005 and between January 1st and December 31st 2014, respectively. Patients who stayed less than 24 h on the NICU or were readmitted after inclusion in the study have been excluded. Follow-up stopped once a patient was discharged or transferred to another ward (e.g., neonatal observation ward, general paediatric wards).

### 2.3. Data Collection

In 2004, demographic data, drug prescriptions and diagnoses of all enrolled patients were documented manually on a daily basis. For the 2014 study, all information was retrieved from the electronic medical records in July 2015. Continuous intravenous infusions, e.g., glucose or sodium chloride, total parenteral nutrition and oxygen administration were excluded. In addition, contrast agents, enemata and all scheduled vaccinations were not included. A positive opinion of the Ethics Committee of the Medical Faculty of the Friedrich-Alexander University Erlangen-Nürnberg has been obtained (reference number 335_14).

### 2.4. Definitions

Definitions for off-label/unlicensed drug use were adopted from Neubert et al. [13] Licensed drug use was defined as the use of a drug with marketing authorisation including information on the neonatal age group (age <1 month) in any available Summary of Product Characteristics (SPC). Off-label use was defined as the use of drugs with marketing authorisation but without any information for neonates found in the SPC. Use of drugs without marketing authorisation in Germany such as imported drugs or drug formulations prepared by the hospital pharmacy was referred to as unlicensed drugs.

### 2.5. Data and Statistical Analysis

The Anatomical Therapeutic Chemical (ATC) classification system was used for the analyses of the prescribed drugs and the International Classification of Diseases, 10th revision (ICD-10) classification system was used for analyses of the recorded diagnoses. Patients were stratified by gestational age: very preterm neonates (24–27 weeks), preterm neonates (28–30 weeks, 31–33 weeks and 34–36 weeks) and term neonates (37 weeks and older). Drug exposure rates were calculated using the number of exposed patients divided by all enrolled patients. Descriptive results are presented with frequencies, means with standard deviations (SD) or median with interquartile range (IQR).

Patient characteristics have been compared to the data collected in the primary study conducted in 2004 [12]. For comparison of the patient characteristics, drug use patterns and off-label/unlicensed use, Chi-square test or Fisher’s exact test were used for categorical variables, as appropriate. For continuous variables, the unpaired two-sample t-test was used. For all statistical tests, a *p*-value < 0.05 was considered statistically significant. Microsoft Excel 2010 and Microsoft Access 2010 (Microsoft Corp., Redmond, WA, USA) were used for data management and IBM SPSS 23 (IBM Corp., Version 23.0. Armonk, NY, USA) was used for the statistical analysis.

## 3. Results

### 3.1. Patient Characteristics

In 2004, a total of 183 (male 55.7%) patients, and in 2014 a total of 204 (male 57.8%) eligible patients, have been admitted to the NICU during the study period. Mean duration of stay on the NICU in 2014 was 21.5 days (2004: 19.4 days). Mean gestational age was 33.6 weeks (SD ± 4.66) in 2004 and 34.1 weeks (SD ± 4.3) in 2014. Preterm neonates were the largest group in both years (2004: n = 100, 54.6%; 2014: n = 115, 56.4%). More details on the study population are given in Table 1.

Comparing the main patient characteristics of the two studies, there were no statistically significant differences (all *p*-values > 0.05) (Table 1).

### 3.2. Drug Prescriptions

In 2014, a total of 2274, and in 2004 a total of 1978 drug prescriptions have been documented respectively. In 2004, this corresponds to 111 (102 evaluated for licensing status) and in 2014 to 102 different drugs. (Table 1) Drugs for the nervous system (2004: 22.6%; 2014: 26.9%) followed by anti-infective drugs for systemic use (2004: 26.0%; 2014: 24.9%), drugs for the cardiovascular system (2014: 14.0%) and drugs for the respiratory system (2004: 15.8%) have been most frequently prescribed in both years. (Table 2) The mean number of prescriptions per patient was, in both studies, 11.1 (2004: SD ± 9.6, max. 45; 2014: SD ± 10.7, max. 63).

### 3.3. Drug Exposure

In 2014, all patients received at least one drug, whereas in 2004 this was the case for 99% of patients. A proportion of 90.7% (2004) and 91.2% (2014) of the patients were treated with at least one anti-infective for systemic use and at least one drug affecting blood and blood forming organs. Drugs for the nervous system received 60.7% (2004) and 70.1% (2014) of the patients, respectively. The percentage of patients treated with drugs acting on the respiratory system decreased from 60.1% in 2004 to 43.6% in 2014. In 2014, more patients were treated with drugs for sensory organs (Table 3).

Overall, patients most frequently received phytomenadione (2004: 89.1%; 2014: 91.2%), piperacillin (2004: 80.3%; 2014: 86.8%) and tobramycin (2004: 79.8%; 2014: 87.3%) in both studies(Table 4 and Table 5).

A significant change in the most frequently used medication was seen in neonates born at GA 24–27 or GA 28–30. While in 2004 caffeine was used for 53.8% and 45.4% of those patients, in 2014 all neonates in those age groups received caffeine. A similar development is seen for cholecalciferol. For the remaining age groups, there was no change in the most frequently used drugs, which were phytomenadione, piperacillin and tobramycin in both years (Table 4 and Table 5).

#### 3.3.1. Anti-Infective Drugs for Systemic Use

Although the overall exposure of anti-infectives was similar (2004: 90.7%; 2014: 91.2%), in 2014 more patients aged 31–33 GA received anti-infectives for systemic use (2004: 82.1%, 2014: 100%) (Table 3).

In 2004, 81.4% (149/183) of all patients received at least one beta lactam antibacterial (J01C) and 79.8% of all patients (n = 146) received aminoglycoside antibiotics. Whereas 86.8% of all patients have been treated with at least one beta lactam antibacterial (J01C) (n = 177/204), each of them piperacillin (n = 177/204) in 2014, 87.3% received at least one aminoglycoside antibiotic, tobramycin (n = 178/204, 87.3%) (Table 4 and Table 5).

In the very preterm age group (24–27 weeks GA), more patients received piperacillin/tobramycin in 2014 (80.0%/86.7% vs. 2004: 61.5%/65.4%). The same applies to the preterm neonates 31–33 weeks GA (20 04: 79.5%; 2014: 100%), (*p* > 0.05). In contrast, in 2004 imipenem/cilastatin was prescribed to 61.5% of very preterm neonates (GA 24–27 weeks) and to 40.9% of those born at GA 28–30 weeks and ceased to be used in 2014 (Table 4 and Table 5).

#### 3.3.2. Central Nervous System Drugs

This anatomical group (ATC: N) accounted for 26.9% (611/2274) of all prescriptions in 2014. Within this group, analgesics (N02) were the drugs prescribed most often (155/611; piritramide 70/155 and metamizole 60/155) followed by psycholeptics (N05) (142/611, midazolam 87/142) and anaesthetics (N01) (119/611) like fentanyl (73/119). In 2004, 448 out of 1978 prescriptions (22.6%) related to CNS drugs. The subgroups of most prescribed drugs just changed their order, psycholeptics (N05: 140/448—midazolam 89/140), followed by anaesthetics (N01: 116/448—fentanyl 74/116) and analgesics (N02: 119/448—piritramide 56/119).

The CNS drug most patients received in 2014 was caffeine (89/204, 43.6%), followed by midazolam (87/204, 42.6%), the antiepileptic phenobarbital (80/204, 39.2%) and the opioids fentanyl (73/204, 35.8%) and piritramide (70/204, 34.3%). In 2004, midazolam (89/183, 48.7%) was most prescribed, followed by the anaesthetic fentanyl (74/183, 40.4%) and the antiepileptic phenobarbital (62/183, 33.8%).

In the age groups GA 28–30 and 31–33 in 2004, caffeine was given to 45.4% (n = 10) and 30.8% (n = 12) of patients only, whereas in 2014, almost each neonate (100%; 94.1%) received this drug. While fentanyl was given to slightly fewerpatients in 2014 (73/204, 35.8%) vs. 2004 (74/183, 40.4%), piritramide was used more often than in 2004 (56/183, 30.6% vs. 2014: 70/204, 34.3%). Particularly in the very preterm age groups, there was an increase in the use of piritramide and fentanyl, whereas a decrease was observed for the older preterm and term neonates, (*p* = <0.05). (Table 4 and Table 5) A similar effect was seen for midazolam: in 2004 midazolam was received less often by younger children but more often by older children than in 2014 (Table 4 and Table 5). Diazepam was only in the age group GA 24–27 weeks and was amongst the most frequently used drugs in 2014, but was neither the most frequently used in other groups nor overall (Table 4 and Table 5).

#### 3.3.3. Cardiovascular System

Overall, there was no change in exposure of this drug group between 2004 and 2014 (44.8% vs. 41.7%). Among diuretics, furosemide is the drug most often used (2014: 29.4%; 2004: 21.3%). However, on the drug level, the use of diuretics like spironolactone and hydrochlorothiazide became more frequent, particularly in very preterm and preterm neonates (Table 4 and Table 5).

### 3.4. Off-Label and Unlicensed Drug Use

In 2014, 39.2% (892/2274) of all prescriptions were off-label and 0.4% (8/2274) were unlicensed, whereas in 2004, 34.3% (678/1978) of all prescriptions were either off-label or unlicensed (Table 1).

**Figure 1:**
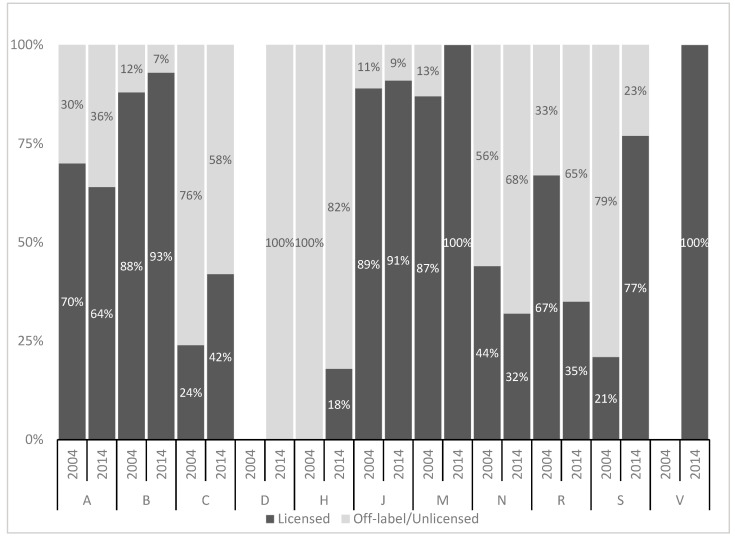
Percentage of prescriptions with or without information on patients aged <1 month in SPCs by ATC anatomical levels (2004 vs 2014). **A:** Alimentary tract and metabolism, **B:** Blood and blood-forming organs, **C:** Cardiovascular system, **D:** Dermatologicals, **H:** Systemic hormonal preparations, excluding sex hormones and insulins, **J:** Anti-infectives for systemic use, **M:** Musculo-skeletal system, **N:** Nervous system, **R:** Respiratory system, **S:** Sensory organs, **V:** Various. No drugs from ATC anatomical levels D and V have been prescribed in the 2004 study.

In 2004, most u/o-prescriptions referred to the drug classes anaesthetics and analgesics. In 2014, analgesic drugs and drugs for obstructive airway diseases/diuretics were prescribed most frequently off-label (Table 6, Figure 1).

In terms of patient exposure, in 2004, 70.0% of all patients received at least one unlicensed/off-label drug and 62.7% in 2014. In both years, 100% of very preterm infants received at least one unlicensed or off-label drug prescription.

On average, unlicensed and off-label use decreased with patient age and varied between 100% and 51.3% (Table 1). In both years, 102 different drugs were assessed with regard to the availability of information for patients less than one month old (licensing status).

In 2004, 62% (n = 63) of them did not have any information regarding the use for patients less than one month old, whereas in 2014, this was the case for 56.9% (n = 58).

For 13 drugs, the licensing status changed either from off-label to label (n = 9) or from label to off-label (n = 4). (Table 7) Changes were particularly observed for anti-infectives and drugs for cardiac therapy.

## 4. Discussion

According to our information, this is the first study comparing the drug utilisation and unlicensed/off-label drug use on an NICU before and after the implementation of the Paediatric Regulation in 2007. We observed similarities, but did also see significant changes in regard to the drugs used and their licensing status. As seen in previous studies, anti-infectives in general and phytomenadione, indicated as prophylaxis for vitamin K deficiency bleeding, were the drugs most frequently prescribed in both years [12,14,15,16].

In our study and the 2004 study, the mean number of drugs per patient was similar (mean 11.1). However, previously published studies show lower numbers varying between 6.4 and 8.8 (mean; median 3–6) [3,16,17,18,19,20,21,22,23,24,25]. The reason for this might be that our NICU is part of a university hospital, which means that more patients with rare diseases and with conceivable difficult outcomes are treated and those patients need a more intensive pharmacotherapy and thus receive more drugs.

The most significant changes in this study were observed in the group of very preterm infants. Whereas in 2004 only about half of this group received caffeine and cholecalciferol, in 2014 the use of these drugs almost doubled and more than 90% of patients received these drugs in 2014. On the other hand, the use of theophylline decreased significantly, particularly in the very preterm groups (24–27 weeks GA and 28–30 weeks GA).

According to the literature, the use of caffeine is better than the use of theophylline due to fewer side effects and a broader therapeutic index. The German Guideline for the therapy of idiopathic apnoeas, bradycardia and hypoxemia in preterm neonates also does not recommend theophylline anymore because much better data is available for caffeine [26,27]. In addition, caffeine became licensed for the treatment of apnoea in newborns in 2009.

This change in standard of care does also explain why in 2014 more central nervous system drugs were prescribed and the percentage of drugs for the respiratory system decreased. Whereas theophylline is part of the latter ATC group (respiratory system), caffeine does belong to the group of central nervous system drugs.

A second significant change in the group of very preterm infants, but also in the group of preterm infants (31–33 weeks GA), was observed in regard to anti-infectives. Whereas imipenem/cilastatin was frequently prescribed in 2004, it was entirely replaced by piperacillin in 2014. One reason for this might be that it showed that the use of imipenem and meropenem is associated with a higher risk for MRSA colonisation than other antibiotics like, e.g., piperacillin [28].

In contrast with the study by Flint et al., the anti-infectives predominantly used were amoxicillin, gentamicin, tobramycin, benzylpenicillin and amoxicillin plus clavulanic acid. It shows that the treatment with antibiotics is different between countries and is related to the experience of the physician, the hospital’s guidelines, or even the cultural circumstances [18,22,23,29,30]. Another reason for the variant antibiotic use is the existence of different bacterial resistances between countries [31].

In terms of analgesics, the drugs used most often in 2004 and 2014 were fentanyl and piritramide. A study by Mehler et al., which compared the analgesic drugs used from 2003–2009 with drug use in 2010 in 46 neonatal units in Germany, found fentanyl, followed by morphine, to be the dominating analgesics. However, piritramide was also frequently used. In line with our results, this study found an increase in the use of analgesic, particularly for very preterm neonates [32]. This can be explained by a change of perception that very preterms do also suffer from pain and should be treated sufficiently with analgesics. Although such guidelines were already published back in 2001, it can be assumed that in 2004 this information was not as present as it was later in 2014 [33]. Interestingly, and this is also in line with Mehler et al., the use of midazolam in the very preterm age groups also increased, despite there being data on its possible negative long-term outcomes. The fact that there is no licensed alternative might explain this [34].

In an international comparison, we could see that in our study, particularly in older patients, metamizole was used increasingly, whereas, e.g., Flint et al., found paracetamol to be the dominating drug. This reflects the fact that metamizole is not on the market in other countries because of a fear of agranulocytosis [35].

Furthermore, we have seen that the drugs which have been used changed considerably within the 10 years. One example of this is anti-infectives. In 2004, imipenem/cilastatin was a frequently used anti-infective, but as already mentioned, was not used in 2014 anymore. Remifentanil, morphine and levetiracetam were the Nervous System Drugs additionally used in 2014, in contrast with tramadol and phenytoin which were no longer in use. Concerning the ATC C, amlodipine and digoxin, which were used in 2004, were not used in 2014 anymore, whereas ibuprofen was established as a new labelled drug for the treatment of patent ductus arteriosus. Nevertheless, from our data, it remains unclear to what extent these changes were purely local changes in standard practice, for instance because of changing local antibiotic resistance, or if they were because of new available evidence.

### 4.1. Licensing Status

Interestingly, the absolute number of off-label prescriptions increased between 2004 (n = 678, 34.3%) and 2014 (n = 900, 39.6%). Flint et al. reported 23% of all prescriptions to be off-label for neonatal age. In this study he also found that the proportion of off-label prescriptions increased with neonatal age, which was not the case in our study. Silva et al. found that 29.2% of prescriptions were off-label for age however, if also considering, for example, dosing, the frequency of administration and indication (use according to SPC) in 57.1% of all prescriptions was found to be off-label. This explains the rather large variety in the proportion of drugs being prescribed off-label in NICU (41.1–73.5%) [24,29,36,37,38]. In our study, we only considered age as an indicator for off-label use. Thus, one can assume that in our results, more prescriptions than were reported were off-label.

The overall exposure rate decreased not significantly from 70% to 62.7%. Flint et al. reported an overall off-label exposure of 54%, whereas Silva reported 69.7%. Other studies also reported similar numbers varying between 48% [39] up to >80% [14,24,29,40]. All studies show that exposure is higher the younger the patients are.

However, looking at the number of drugs considered in 2004, more than half (62%) of them did not have any information regarding their use in patients less than one month old. In 2014, this was the case for 56.9% of drugs. In our analysis, the SPC was changed for nine drugs in terms of their use in term and preterm neonates that now can be considered as being on a“label”. On the other hand, four drugs (e.g., theophylline and acetylcysteine) now have to be considered as off-label, which was not the case earlier.

This may suggest that new data regarding neonatal drug use is becoming available and this is reflected in the SPCs. The fact that there is not only a change from off-label to label but also vice versa, confirms that labels do not automatically mean the best evidence is given. This particularly concerns older drugs, which were licensed with much lower requirements than they are in place now.

However, the number of drugs that have become licensed remains low. This might be one of the meanwhile well-known problems of the paediatric regulation. Whereas a paediatric investigation plan is mandatory for new drugs, there is no obligation for drugs which are already on the market and which do not have a patent protection anymore. There is little interest for pharma companies to develop these drugs for the paediatric population because the profit is too low compared to the costs of the studies needed [9].

Nevertheless, these findings have to be interpreted with care. Firstly, the availability of a given formulation is based on the manufacturer’s discretion, so our setting in a German neonatal unit does not necessary reflect the setting in other EU countries. Secondly, off-label use does not necessarily mean off-evidence use. The availability of evidence for the use of a drug is what is most important and needs to distinguish between the use of a drug if evidence suggests that it is not efficacious or may cause harm or if there is evidence suggesting that the use of this drug will be of therapeutic benefit for the patient. Unfortunately, off-label prescribing is not regulated by European law and there is heterogeneity among countries with regard to legal and economicaspects of off-label use. Although, with some restrictions, it is often ethically and legally accepted that there are a lot of uncertainties related to reimbursement practices. A recent policy statement by the European Academy of Paediatrics and the European Society for Developmental Perinatal and Paediatric Pharmacology highlights the various aspect to be considered when prescribing medicines off-label and provides guidance to Health Care Professionals with regard to prescribing off-label medicines [41].

### 4.2. Strength and Limitations

This study provides a comprehensive overview of paediatric drug utilisation. Particularly, data from 2014 shows a high validity as it has been extracted from the electronic patient record. Both datasets have been assessed manually with regards to licensing status.

Our data have, however, not been adjusted by diagnosis or treatments (e.g., surgical, non-surgical) and dosing regimens were not considered. Prescribing trends therefore have to be interpreted with care. In addition, in terms of the licensing status, we only considered whether the neonatal age group was present in the SPC but did not refer to indications and dosing regimen. This may have led to an underestimation of off-label drug use.

In addition, we may have underestimated the amount of unlicensed used drug as only medicinal products prepared by the hospital pharmacy were taken into consideration. Bedside preparation was not taken into account due to the retrospective nature of the study. Nevertheless, these kinds of drugs are being captured as off-label and thus the overall rates should be realistic numbers.

Finally, this study is a single centre study conducted in Germany. Our findings confirm similar reports but do not provide a broader European or international setting.

## 5. Conclusions

This study provides a 10-year comparison of drug utilisation in a German NICU setting. Overall, there was no significant change neither in terms of the drugs used nor regarding their licensing status. However, new data became available resulting in changes in SPCs. This may mean that in the first six years following the introduction of the paediatric regulation in Europe new evidence has been introduced to neonatal drug therapy although, this would only have happened to a small extent. However, this study only reflects the German neonatal setting. Further multi-centre studies across Europe are needed to validate these results.

## Figures and Tables

**Table 1:** Patient characteristics.

	Very Preterm Neonates	Preterm Neonates			Term Neonates	Total
	GA 24–27 Weeks	GA 28–30 Weeks	GA 31–33 Weeks	GA 34–36 Weeks	GA ≥ 37 Weeks	
	2004	2004	2004	2004	2004	2004
	2014	2014	2014	2014	2014	2014
Number of patients (n, %)	26 (14.2) 15 (7.4)	22 (12.0) 22 (10.8)	39 (21.3) 34 (16.7)	39 (21.3) 59 (28.9)	57 (31.1) 74 (36.3)	183 (100.0) 204 (100.0)
Gender (male; n, %)	14 (53.8) 8 (53.3)	11 (50.0) 13 (59.1)	24 (61.5) 19 (55.9)	18 (46.2) 34 (57.6)	35 (61.4) 44 (59.5)	102 (55.7) 118 (57.8)
Weight (gram; mean ± SD)	830 ± 248.9 745 ± 217	1372 ± 430.6 1060 ± 252	1777 ± 438.7 1679 ± 317	2431 ± 609.1 2223 ± 488	3065 ± 556.5 3040 ± 662	2134 ± 935 2195 ± 923
Length of hospital stay (days; median, IQR, max)	38.8 (36.0, 121.0) 69.3 (110.2, 252.0)	25.0 (22.0, 120.0) 65.5 (40.0, 247.1)	13.0 (8.0, 87.0) 35.2 (14.8, 230.4)	17.5 (9.0, 105.0) 16.6 (13.7, 135.1)	13.9 (9.0, 167.0) 8.8 (19.1, 148.1)	19.4 (11, 167) 20.8 (34.2, 252.0)
Survival (n, %)	25 (96.2) 14 (93.3)	22 (100) 22 (100)	36 (92.3) 34 (100)	36 (92.3) 59 (100)	55 (96.5) 73 (98.6)	174 (95.1) 202 (99.0)
Patients with at least one OL/UL drug (n, %)	26 (100.0) 15 (100.0)	19 (86.4) 20 (90.9)	20 (51.3) 20 (58.8)	24 (61.5) 32 (54.2)	39 (68.4) 41 (55.4)	128 (70.0) 128 (62.7)
Number of different drugs (n)/ different drugs OL/UL	78/41 70/40	43/19 66/37	56/24 60/32	65/32 69/33	76/40 81/46	111 */63 102/58
Number of prescriptions (n, %)	498 (25.2) 333 (14.6)	244 (12.3) 429 (18.9)	278 (14.1) 363 (16.0)	387 (19.6) 483 (21.2)	571 (28.9) 666 (29.3)	1978 (100.0) 2274 (100.0)
Number of all prescriptions OL/UL (n, %)	207 (41.6) 154 (46.2)	59 (24.2) 170 (39.6)	73 (26.3) 112 (30.9)	130 (33.6) 174 (36.0)	209 (36.6) 290 (43.5)	678 (34.3) 900 (39.6)

GA: gestational age; OL: off-label; UL: unlicensed; * for 9 drugs the licensing status could not be identified.

**Table 2:** Frequency of prescriptions by ATC (Anatomical Therapeutic Chemical) Classification in 2004/2014.

	Very Preterm Neonates	Preterm Neonates			Term Neonates	
	GA 24–27 Weeks	GA 28–30 Weeks	GA 31–33 Weeks	GA 34–36 Weeks	GA ≥ 37 Weeks	Total
	n (%)	n (%)	n (%)	n (%)	n (%)	n (%)
	2004	2004	2004	2004	2004	2004
	2014	2014	2014	2014	2014	2014
Alimentary tract and metabolism	37 (7.4) 26 (7.8)	19 (7.8) 41 (9.6)	29 (10.4) 32 (8.8)	36 (9.3) 35 (7.2)	47 (8.2) 55 (8.3)	168 (8.5) 189 (8.3)
Blood and blood forming organs	31 (6.2) 19 (5.7)	23 (9.4) 39 (9.1)	47 (16.9) 42 (11.6)	45 (11.6) 64 (13.3)	68 (11.9) 84 (12.6)	214 (10.8) 248 (10.8)
Cardiovascular system	62 (12.4) 58 (17.4)	16 (6.6) 72 (16.8)	23 (8.3) 37 (10.2)	29 (7.5) 61 (12.6)	57 (10.0) 91 (13.7)	187 (9.5) 319 (14.0)
Dermatologicals	1 (0.2) 4 (1.2)	1 (0.4) 4 (0.9)	- 5 (1.4)	1 (0.3) 1 (0.2)	1 (0.2) 1 (0.2)	4 (0.2) 15 (0.7)
Systemic hormonal preparations, excluding sex hormones and insulins	13 (2.6) 13 (3.9)	- 10 (2.3)	2 (0.7) 3 (0.8)	1 (0.3) 4 (0.8)	6 (1.1) 8 (1.2)	22 (1.1) 38 (1.7)
Anti-infectives for systemic use	114 (22.9) 70 (21.0)	76 (31.1) 89 (20.7)	76 (27.3) 93 (25.6)	103 (26.6) 135 (28.0)	145 (25.4) 180 (27.0)	514 (26.0) 567(24.9)
Musculo-skeletal system	21 (4.2) 11 (3.3)	10 (4.1) 11 (2.6)	8 (2.9) 10 (2.8)	16 (4.1) 15 (3.1)	23 (4.0) 16 (2.4)	78 (3.9) 63 (2.8)
Nervous system	122 (24.5) 93 (27.9)	34 (13.9) 112 (26.1)	39 (14.0) 96 (26.4)	98 (25.3) 131 (27.1)	155 (27.1) 179 (26.9)	448 (22.6) 611 (26.9)
Antiparasitic products, insecticides and repellents	- -	- -	- -	1 (0.3) -	- -	1 (0.05) 0
Respiratory system	88 (17.7) 29 (8.7)	63 (25.8) 31 (7.2)	47 (16.9) 32 (8.8)	51 (13.2) 31 (6.4)	63 (11.0) 44 (6.6)	312 (15.8) 167 (7.3)
Sensory organs	8 (1.6) 9 (2.7)	2 (0.8) 18 (4.2)	7 (2.5) 12 (3.3)	3 (0.8) 3 (0.6)	4 (0.7) 6 (0.9)	24 (1.2) 48 (2.1)
Various	1 (0.2) 1 (0.3)	- 2 (0.5)	- 1 (0.3)	3 (0.8) 3 (0.6)	2 (0.4) 2 (0.3)	6 (0.3) 9 (0.4)
**Total in 2004 (%)**	**498 (25.2)**	**244 (12.3)**	**278 (14.1)**	**387 (19.6)**	**571 (28.9)**	**1978 (100)**
**Total in 2014 (%)**	**333 (14.6)**	**429 (18.9)**	**363 (16.0)**	**483 (21.2)**	**666 (29.3)**	**2274 (100)**

n = number of prescriptions.

**Table 3:** Exposure rate (%) and number of patients receiving at least one drug (ATC chemical level) by gestational age, 2004 vs 2014.

	ATC	Very Preterm Neonates 2004	Very Preterm Neonates 2014	Preterm Neonates 2004			Preterm Neonates 2014			Term Neonates 2004	Term Neonates 2014	Total 2004	Total 2014
		GA 24–27 Weeks	GA 24–27 Weeks	GA 28–30 Weeks	GA 31–33 Weeks	GA 34–36 Weeks	GA 28–30 Weeks	GA 31–33 Weeks	GA 34–36 Weeks	GA 24–27 Weeks	GA ≥ 37 Weeks	N= 183	N = 204
		N = 26	N = 15	N = 22	N = 39	N = 39	N = 22	N = 34	N = 59	N = 57	N = 74		
A	Alimentary tract and metabolism	76.9	93.3	72.7	41.0	48.7	100.0	52.9	28.8	45.6	39.2	53.0	49.0
		(n = 20)	(n = 14)	(n = 16)	(n = 16)	(m = 19)	(n = 22)	(n = 18)	(n = 17)	(n = 26)	(n = 29)	(n = 97)	(n = 100)
B	Blood and blood-forming organs	69.2	66.7	100	94.9	92.3	95.5	97.1	94.9	93.0	89.2	90.7	91.2
		(n = 18)	(n = 10)	(n = 22)	(n = 37)	(n = 36)	(n = 21)	(n = 33)	(n = 56)	(n = 53)	(n = 66)	(n = 166)	(n = 186)
C	Cardiovascular system	88.5	86.7	50.0	30.8	28.2	77.3	38.2	23.7	43.9	37.8	44.8	41.7
		(n = 23)	(n = 13)	(n = 11)	(n = 12)	(n = 11)	(n = 17)	(n = 13)	(n = 14)	(n = 25)	(n = 28)	(n = 82)	(n = 85)
D	Dermatologicals	-	26.7	-	-	-	18.2	8.8	1.7	-	1.4	-	6.4
			(n = 4)				(n = 4)	(n = 3)	(n = 1)		(n = 1)		(n = 13)
H	Systemic hormonal preparations, excluding aex hormones and insulins	34.6 (n = 9)	46.7 (n = 7)	-	5.1 (n = 2)	2.6 (n = 1)	27.3 (n = 6)	5.9 (n = 2)	5.1 (n = 3)	8.8 (n = 5)	9.5 (n = 7)	9.3 (n = 17)	12.3 (n = 25)
J	Anti-infectives for systemic use	96.2	100.0	100	82.1	84.6	95.5	100.0	88.1	94.7	86.5	90.7	91.2
		(n = 25)	(n = 15)	(n = 22)	(n = 32)	(n = 33)	(n = 21)	(n = 34)	(n = 52)	(n = 54)	(n = 64)	(n = 166)	(n = 186)
M	Musculoskeletal system	69.2	66.7	40.9	20.5	38.5	45.5	29.4	25.4	38.6	18.9	39.3	28.9
		(n = 18)	(n = 10)	(n = 9)	(n = 8)	(n = 15)	(n = 10)	(n = 10)	(n = 15)	(n = 22)	(n = 14)	(n = 72)	(n = 59)
N	Nervous system	96.2	100.0	54.5	35.9	56.4	100.0	97.1	57.6	66.7	52.7	60.7	70.1
		(n = 25)	(n = 15)	(n = 12)	(n = 14)	(n = 22)	(n = 22)	(n = 33)	(n = 34)	(n = 38)	(n = 39)	(n = 111)	(n = 143)
R	Respiratory system	84.6	60.0	90.9	48.7	56.4	63.6	47.1	37.3	47.4	37.8	60.1	43.6
		(n = 22)	(n = 9)	(n = 20)	(n = 19)	(n = 22)	(n = 14)	(n = 16)	(n = 22)	(n = 27)	(n = 28)	(n = 110)	(n = 89)
S	Sensory organs	23.1	53.3	9.1	10.3	5.1	59.1	29.4	5.1	7.0	6.8	9.8	19.1
		(n = 6)	(n = 8)	(n = 2)	(n = 4)	(n = 2)	(n = 13)	(n = 10)	(n = 3)	(n = 4)	(n = 5)	(n = 18)	(n = 39)
V	Various	-	6.7	-	-	-	9.1	2.9	5.1	-	2.7	-	4.4
			(n = 1)				(n = 2)	(n = 1)	(n = 3)		(n = 2)		(n = 9)

**Table 4:** Number and percentage of the most-prescribed drugs (ATC chemical level) by gestational age in 2014.

Very Preterm Neonates			Preterm Neonates									Term Neonates			Total		
GA 24–27 Weeks			GA 28–30 Weeks			GA 31–33 Weeks			GA 34–36 Weeks			GA ≥ 37 Weeks					
N = 15	n	%	N = 22	n	%	N = 34	n	%	N = 59	n	%	N = 74	n	%	N = 204	n	%
Caffeine	15	100	Caffeine	22	100	Tobramycin	34	100	Phytomenadione	56	94.9	Phytomenadione	66	89.2	Phytomenadione	186	91.2
Cholecalciferol	14	93.3	Cholecalciferol	22	100	Piperacillin	34	100	Tobramycin	50	84.7	Tobramycin	60	81.1	Tobramycin	178	87.3
Midazolam	13	86.7	Tobramycin	21	95.5	Phytomenadione	33	97.1	Piperacillin	50	84.7	Piperacillin	60	81.1	Piperacillin	177	86.8
Piritramide	13	86.7	Piperacillin	21	95.5	Caffeine	32	94.1	Theophylline	19	32.2	Midazolam	31	41.9	Caffeine	89	43.6
Tobramycin	13	86.7	Phytomenadione	21	95.5	Cholecalciferol	17	50.0	Midazolam	19	32.2	Phenobarbital	26	35.1	Midazolam	87	42.6
Piperacillin	12	80.0	Ferrous glycine sulfate	17	77.3	Theophylline	13	38.2	Phenobarbital	19	32.2	Piritramide	25	33.8	Phenobarbital	80	39.2
Fentanyl	11	73.3	Phenobarbital	15	68.2	Midazolam	12	35.3	Fentanyl	19	32.2	Metamizole sodium	23	31.1	Fentanyl	73	35.8
Furosemide	10	66.7	Piritramide	13	59.1	Phenobarbital	10	29.4	Caffeine	18	30.5	Fentanyl	22	29.7	Piritramide	70	34.3
Phenobarbital	10	66.7	Tropicamide	13	59.1	Fentanyl	10	29.4	Vecuronium	14	23.7	Furosemide	21	28.4	Cholecalciferol	65	31.9
Phytomenadione	10	66.7	Midazolam	12	54.5	Vecuronium	10	29.4	Furosemide	13	22.0	Sodium fluoride, Combinations	17	23.0	Theophylline	63	30.9
Vecuronium	10	66.7	Dobutamine	12	54.5	Metamizole sodium	10	29.4	Metamizole sodium	12	20.3	Theophylline	16	21.6	Metamizole sodium	60	29.4
Ferrous glycine sulfate	9	60.0	Hydrochloro-thiazide	12	54.5	Ferrous glycine sulfate	9	26.5	Sodium fluoride, Combinations	11	18.6	Omeprazole	16	21.6	Furosemide	60	29.4
Ibuprofen	9	60.0	Spironolactone	12	54.5	Piritramide	9	26.5	Piritramide	10	16.9	Epinephrine	15	20.3	Vecuronium	58	28.4
Vancomycin	9	60.0	Fentanyl	11	50.0	Tropicamide	9	26.5	Dobutamine	9	15.3	Vecuronium	14	18.9	Dobutamine	44	21.6
Dobutamine	8	53.3	Natural phospholipids	11	50.0	Natural phospholipids	8	23.5	Norepinephrine	9	15.3	Norepinephrine	13	17.6	Ferrous glycine sulfate	44	21.6
Hydrochloro-thiazide	8	53.3	Furosemide	10	45.5	Metronidazole	7	20.6	Omeprazole	8	13.6	Vancomycin	12	16.2	Vancomycin	43	21.1
Spironolactone	8	53.3	Vecuronium	10	45.5	Dobutamine	6	17.6	Cholecalciferol	7	11.9	Chloral hydrate	12	16.2	Epinephrine	40	19.6
Tropicamide	8	53.3	Vancomycin	9	40.9	Hydrochloro-thiazide	6	17.6	Natural phospholipids	7	11.9	Oxymetazoline	12	16.2	Norepinephrine	37	18.1
Diazepam	7	46.7	Palivizumab	9	40.9	Spironolactone	6	17.6	Metronidazole	7	11.9	Metronidazole	11	14.9	Metronidazole	37	18.1
Epinephrine	7	46.7	Epinephrine	8	36.4	Furosemide	6	17.6	Vancomycin	7	11.9	Esketamine	11	14.9	Tropicamide	37	18.1
Meropenem	7	46.7	Meropenem	8	36.4	Vancomycin	6	17.6	Potassium canrenoate	7	11.9	Heparin	11	14.9	Spironolactone	36	17.6

N: number of patients in age group; n: number of patients exposed to a drug; %: exposure rate to a drug in that age group.

**Table 5:** Number and percentage of most prescribed drugs (ATC chemical level) by gestational age in 2004.

Very Preterm Neonates			Preterm Neonates									Term Neonates			Total		
GA 24–27 Weeks			GA 28–30 Weeks			GA 31–33 Weeks			GA 34–36 Weeks			GA ≥ 37 Weeks					
N = 26	n	%	N = 22	n	%	N = 39	n	%	N = 39	n	%	N = 57	n	%	N = 183	n	%
Midazolam	21	80.8	Phytomenadione	22	100	Phytomenadione	37	94.9	Phytomenadione	36	92.3	Phytomenadione	52	91.2	Phytomenadione	163	89.1
Phenobarbital	20	76.9	Piperacillin	22	100	Piperacillin	31	79.5	Piperacillin	31	79.5	Piperacillin	47	82.5	Piperacillin	147	80.3
Vancomycin	20	76.9	Tobramycin	22	100	Tobramycin	31	79.5	Tobramycin	31	79.5	Tobramycin	45	78.9	Tobramycin	146	79.8
Theophylline	19	73.1	Theophylline	19	86.4	Theophylline	16	41.0	Midazolam	19	48.7	Midazolam	29	50.9	Theophylline	95	51.9
Fentanyl	17	65.4	Cholecalciferol	14	63.3	Caffeine	12	30.8	Theophylline	18	46.2	Fentanyl	24	42.1	Midazolam	89	48.6
Tobramycin	17	65.4	Surfactant	11	50.0	Cholecalciferol	12	30.8	Fentanyl	16	41.0	Theophylline	23	40.3	Fentanyl	74	40.4
Vecuronium	17	65.4	Caffeine	10	45.4	Dobutamine	11	28.2	Vecuronium	15	38.5	Phenobarbital	21	36.8	Vecuronium	68	37.2
Dobutamine	16	61.5	Vancomycin	10	45.5	Midazolam	11	28.2	Piritramide	14	35.9	Vecuronium	21	36.8	Phenobarbital	62	33.9
Imipenem and Cilastatin	16	61.5	Imipenem and Cilastatin	9	40.9	Fentanyl	10	25.6	Metamizole sodium	13	33.3	Metamizole sodium	20	35.1	Vancomycin	59	32.2
Phytomenadione	16	61.5	Midazolam	9	40.9	Vecuronium	8	20.5	Omeprazole	11	28.2	Sodium fluoride, Combinations	20	35.1	Dobutamine	58	31.7
Piperacillin	16	61.5	Dobutamine	8	36.4	Surfactant	7	17.9	Vancomycin	11	28.2	Diazepam	17	29.8	Piritramide	56	30.6
Piritramide	16	61.5	Phenobarbital	8	36.4	Piritramide	5	12.8	Sodium fluoride, Combinations	10	25.6	Furosemide	17	29.8	Diazepam	47	25.7
Diazepam	15	57.7	Acetylcysteine	7	31.8	Epinephrine	4	10.3	Phenobarbital	10	25.6	Piritramide	17	29.8	Cholecalciferol	46	25.1
Caffeine	14	53.8	Fentanyl	7	31.8	Heparin	4	10.3	Furosemide	9	23.1	Dobutamine	15	26.3	Imipenem and Cilastatin	45	24.6
Cholecalciferol	14	53.8	Vecuronium	7	31.8	Imipenem amd Cilastatin	4	10.3	Diazepam	8	20.5	Omeprazole	15	26.3	Metamizole sodium	43	23.5
Fluconazole	13	50.0	Oxymetazoline	6	27.3	Sodium fluoride, Combinations	4	10.3	Dobutamine	8	20.5	Vancomycin	14	24.6	Sodium fluoride, Combinations	40	21.9
Spironolactone	12	46.2	Diazepam	4	18.2	Nystatin	4	10.3	Esketamine	8	20.5	Esketamine	12	21.0	Furosemide	39	21.3
Acetylcysteine	11	42.3	Fluconazole	4	18.2	Omeprazole	4	10.3	Metronidazole	8	20.5	Imipenem and Cilastatin	9	15.8	Caffeine	38	20.8
Hydrochloro-thiazide	11	42.3	Ipratropium bromide	4	18.2	Oxymetazoline	4	10.3	Acetylcysteine	7	17.9	Ipratropium bromide	9	15.8	Surfactant	35	19.1
Ipratropium bromide	11	42.3	Piritramide	4	18.2	Vancomycin	4	10.3	Imipenem & Cilastatin	7	17.9	Paracetamol	9	15.8	Omeprazole	35	19.1
Salbutamol	11	42.3	Salbutamol	4	18.2	Diazepam	3	7.7	Oxymetazoline	7	17.9	Salbutamol	9	15.8	Acetylcysteine	33	18.0

N: number of patients in age group; n: number of patients exposed to a drug; %: exposure rate to a drug in that age group.

**Table 6:** Drug groups with most unlicensed/off-label prescriptions 2004 vs 2014.

ATC		2004			2014		
		No of u/o Drugs/Total No of Drugs	No of u/o Prescriptions/Total No of Prescriptions	% u/o Prescriptions	No of u/o Drugs/Total No of Drugs	No of u/o Prescriptions/Total No of Prescriptions	Percentage of o/u Prescriptions
Drugs for acid-related disorders	A02	1/1	35/35	100	2/2	36/36	100
Drugs used in diabetes	A10	3/3	6/6	100	1/1	8/8	100
Antithrombotic agents	B01	4/5	18/26	69.2	3/3	17/17	100
Cardiac therapy	C01	2/6	67/101	66.3	4/9	61/133	45.9
Diuretics	C03	3/4	71/81	87.6	3/5	102/165	61.8
Corticosteroids for systemic use	H02	5/5	22/22	100	3/3	30/30	100
Antibacterials for systemic use	J01	7/15	51/478	10.67	6/15	49/533	9.2
Antiinflammatory and antirheumatic products	M01	2/2	10/10	100	0/1	0/5	0
Anesthetics	N01	6/6	116/116	100	5/5	119/119	100
Analgetics	N02	4/4	119/119	100	4/5	135/155	87.1
Antiepileptics	N03	3/4	11/73	15.1	3/3	106/106	100
Respiratory system drugs	R	5/11	104/312	33.3	6/9	109/167	65.3
Ophthalmologicals	S01	5/6	19/24	79.2	1/2	11/48	22.9

**Table 7:** Change of licensing status regarding the age, 2004 to 2014.

Drug	2004	2014	Comment
Amphotericin B	Off-label	Label	
Caffeine	Off-label	Label	
Dobutamine	Off-label	Label	
Ibuprofen	Off-label	Label	new indication licensed (C01EB16)
Indomethacin	Off-label	Label	not used in 2014
Metronidazole	Off-label	Label	
Paracetamol	Off-label	Label	used as an analgesic (N02BE01)
Spironolactone	Off-label	Label (newborn) Off-label (prematurely born)	change of licensing status in term neonates
Ursodesoxycholic acid	Off-label	Label	
Acetylcysteine	Label	Off-label	
Epinephrine	Label	Off-label	
Silicones	Label	Off-Label	
Theophylline	Label	Off-label	

## Availability of Data and Material (Data Transparency)

The datasets generated during and/or analysed during the current study are available from the corresponding author on reasonable request.
